# Information-theoretic sensitivity analysis: a general method for credit assignment in complex networks

**DOI:** 10.1098/rsif.2007.1079

**Published:** 2007-06-26

**Authors:** Niklas Lüdtke, Stefano Panzeri, Martin Brown, David S Broomhead, Joshua Knowles, Marcelo A Montemurro, Douglas B Kell

**Affiliations:** 1 School of Chemistry, The University of Manchester 131 Princess Street, Manchester M1 7DN, UK; 2 The Manchester Interdisciplinary Biocentre, The University of Manchester 131 Princess Street, Manchester M1 7DN, UK; 3 Faculty of Life Sciences, The University of Manchester 131 Princess Street, Manchester M1 7DN, UK; 4 School of Electrical and Electronic Engineering, The University of Manchester 131 Princess Street, Manchester M1 7DN, UK; 5 School of Mathematics, The University of Manchester 131 Princess Street, Manchester M1 7DN, UK; 6 School of Computer Science, The University of Manchester 131 Princess Street, Manchester M1 7DN, UK

**Keywords:** sensitivity analysis, information theory, entropy, mutual information, communication channel, NFκB

## Abstract

Most systems can be represented as networks that couple a series of nodes to each other via one or more edges, with typically unknown equations governing their quantitative behaviour. A major question then pertains to the importance of each of the elements that act as system inputs in determining the output(s). We show that any such system can be treated as a ‘communication channel’ for which the associations between inputs and outputs can be quantified via a decomposition of their mutual information into different components characterizing the main effect of individual inputs and their interactions. Unlike variance-based approaches, our novel methodology can easily accommodate correlated inputs.

## 1. Introduction

The analysis of networks represents a crucial focus of modern systems biology (Barabási & Oltvai 2004; Hwang *et al*. 2005; Kitano *et al*. 2005; Klipp *et al*. 2005; Wagner 2005; Alon 2006; Davidson 2006; Doyle & Stelling 2006; Kell 2006*a*,*b*; Palsson 2006). For many areas of interest, models of complex networks can be taken to have the form of a deterministic mapping from a set of *n* inputs to one or more output(s) (figure 1). The outputs can be considered separately so that for each output *Y*_*k*_ there is a map

$$f_{k}:(X_{1},\ldots ,X_{n})\mapsto Y_{k}.$$

Usually, the input–output mapping is not available in explicit form but can be evaluated numerically for any given inputs.

Global sensitivity analysis aims to rank the inputs *X*_1_, …, *X*_*n*_ according to the degree to which they influence the output, individually and conjointly. Here, ‘inputs’ may also refer to intrinsic model parameters whose influence on the output is to be determined as in figure 1*b*. This type of global sensitivity analysis is commonly performed in a probabilistic manner by evaluating the model for multiple sets of randomly and independently selected input values drawn, for instance, from uniform distributions over suitable intervals. The output, being a function of the randomized inputs, thus also becomes a random variable. If the inputs are sampled independently, the variance of the output distribution can be decomposed into contributions by individual inputs, pairs, triplets and so forth. This procedure is well known in statistics as ‘analysis of variance’ (ANOVA; e.g. Box *et al*. 1978), and several authors have contributed to improve its computational efficiency for sensitivity analysis (e.g. Rabitz & Aliş 1999; Sobol 2001).

Rather than analysing the *variance* of the output distribution, we take a different route measuring output uncertainty in terms of Shannon's entropy (Shannon & Weaver 1949). Our starting point is the concept of the ‘communication channel’ (Cover & Thomas 2006), which enables us to view the model as a transmitter of information between inputs and outputs (figure 1*b*).

The *mutual information* of two variables is a quantity that measures their mutual dependence (Cover & Thomas 2006). Determining the mutual information *I*(*X*_*i*_;*Y*) between random sampling sequences of individual inputs *X*_*i*_ and their output counterpart can elucidate first-order input–output relations. Mutual information provides a general measure of association that is applicable regardless of the shape of the underlying distributions and—unlike linear- or rank-order correlation—insensitive to non-monotonic dependence among the random variables. Further insight can be obtained by unravelling *conditional dependencies* among the system inputs. Here, we define novel and general sensitivity measures of second and higher order by evaluating input correlations induced by conditioning on the output. To our knowledge, only a first-order information-based analysis has been discussed in the literature to date (Critchfield *et al*. 1986; Dalle Molle & Morris 1993, pp. 402–407).

While variance adequately quantifies the variability of distributions that are symmetrical and unimodal, entropy is calculated directly from the probability distribution function and thus provides a more general measure of output variability. Therefore, we further develop an information-theoretic framework for the sensitivity measures thus derived, based on the observation that their sum is bounded from above by the output entropy *H*(*Y*). From this viewpoint, the (information-theoretic) sensitivity indices quantify the amount of output uncertainty removed by the knowledge of individual inputs and combinations thereof.

Sensitivity analysis of this kind is also an analysis of the *total* mutual information *I*(*X*_1_, *…*,*X*_*n*_;*Y*), which subsumes *all* input–output associations including interactions. The resultant summation theorem for the sensitivity measures is an information balance in which the sum equals *I*(*X*_1_, *…*, *X*_*n*_;*Y*). Although in practice only effects of up to third- or fourth-order can easily be calculated explicitly, the joint impact of all higher order terms is provided by the remaining difference to *I*(*X*_1_, *…*, *X*_*n*_;*Y*). We can therefore assign credit or influence fully to all the parameters of a system over a wide range of operating conditions.

For all variance-based approaches, the absence of input correlations is a critical prerequisite for the uniqueness of the variance decomposition (Saltelli *et al*. 2000, 2004). As will be demonstrated in our methodology, independent inputs merely simplify the analysis. If input correlations exist (e.g. due to non-orthogonal sampling), their effect can easily be taken into account. We apply the methodology successfully to a model of the NFκB signalling pathway and thereby define how to modify its behaviour to provide a designed maximum effect.

## 2. Methods

By randomly sampling the input space, a genuinely deterministic system can be analysed in stochastic terms. Random perturbation of the inputs creates a randomized output *Y* with a probability density *p*(*y*). Rather than attempting to find some parametric model of *p*(*y*), the output density is approximated by a histogram, and the output becomes a *discrete* random variable. The corresponding entropy

(2.1) $$H(Y)=-\sum_{y}p(y){\text{log}}_{2}\;p(y)$$

measures a (hypothetical) receiver's uncertainty about *Y* due to input perturbation. For instance, if one input *X*_*i*_ is fixed, the receiver's remaining uncertainty can be quantified by the *conditional* entropy

(2.2) $$H(Y|X_{i})=\sum_{x}p(x)H(Y|X_{i}=x),$$

which is the average uncertainty in *Y* over all possible discrete values *x* that the input variable *X*_*i*_ can assume. The discretization of *X*_*i*_ and *Y* is, of course, arbitrary and should be chosen in relation to the number of system evaluations (simulation runs).

The mutual information is defined as the difference in output uncertainty with and without knowledge of *X*_*i*_, and characterizes the influence *X*_*i*_ exerts on *Y*:

(2.3) $$I(X_{i};Y)=H(Y)-H(Y|X_{i}).$$

The link between uncertainty and association established by equation (2.3) is one of the fundamental concepts of Shannon's information theory and forms the basis of our framework for sensitivity analysis. Calculating the mutual information *I*(*X*_*i*_;*Y*) for each *X*_*i*_ constitutes a form of first-order sensitivity analysis, assessing only the influence of individual inputs.

### 2.1 An information-theoretic first-order sensitivity index

Critchfield *et al*. (1986) defined the *mutual information index* (MII), which in our notation is the mutual information normalized by the entropy of the output variable:

(2.4) $$s_{i}=\frac{I(X_{i};Y)}{H(Y)}.$$

A first-order sensitivity analysis can be performed by calculating the MII of all inputs, where the mutual information is obtained by computing

(2.5) $$I(X_{i};Y)=\sum_{x_{i}}\sum_{y}p(x_{i},y){\text{log}}_{2}\frac{p(x_{i},y)}{p(x_{i})p(y)}.$$

Though *X*_*i*_ and *Y* are continuous variables, equation (2.5) contains discrete sums, indicating that, in practice, the probability densities are evaluated via the joint histogram and the marginal histograms of the input and output sequences.

### 2.2 Pairwise interactions

If we assume that, by design of the simulation, random input values are drawn independently, there will be no *a priori* correlations among the sequences of input values. However, if inputs interact in their influence on an output, one would expect to find associations in input sequences when conditioning on a particular value of that output. We show that the output-induced conditional dependence among two inputs, characterized by the *conditional mutual information*

(2.6) $$I(X_{i},X_{j}|Y)=\sum_{y}p(y)\sum_{x_{i},x_{j}}p(x_{i},x_{j}|y){\text{log}}_{2}\frac{p(x_{i},x_{j}|y)}{p(x_{i}|y)p(x_{j}|y)},$$

provides a measure of the joint influence of the pair (*X*_*i*_, *X*_*j*_) on the output *Y*, on average.

To understand why this is indeed an appropriate measure, we consider the degree of association among (*X*_*i*_,*X*_*j*_) and *Y*, that is the mutual information *I*(*X*_*i*_,*X*_*j*_*;Y*). Since this quantity subsumes first- and second-order effects, one has to subtract the influence of the individual inputs, *I*(*X*_*i*_*;Y*) and *I*(*X*_*j*_*;Y*), in order to obtain the pure second-order effect of *X*_*i*_ and *X*_*j*_ on *Y*. Using an auxiliary formula proved in appendix A.3, one obtains

(2.7) $$\underset{\text{first}+\text{second}-\text{order}}{\underset{︸}{I(X_{i},X_{j};Y)}}-\underset{\text{first}-\text{order}}{\underset{︸}{I(X_{i};Y)-I(X_{j};Y)}}=\underset{\text{second}-\text{order}}{\underset{︸}{I(X_{i};X_{j}|Y)}}-\underset{\text{input}\;\text{correlation}}{\underset{︸}{I(X_{i},X_{j})}}.$$

The second term on the right-hand side subtracts the effect of any *a priori* input associations due to the applied sampling scheme. If inputs are sampled independently, the term vanishes and the conditional mutual information by itself captures the joint effect of *X*_*i*_ and *X*_*j*_ on *Y*. Note, the simple structure of equation (2.7) makes it possible to apply arbitrary input sampling schemes, without having to be concerned about statistical independence. This reveals a considerable advantage of the information-theoretic approach over variance-based methods, which are not easily extended to non-orthogonal samples (Saltelli *et al*. 2000).

### 2.3 Higher order interactions

Capturing interactions among three or more inputs in information-theoretic terms requires generalizing the concept of mutual information beyond two variables. To characterize the genuine three-way interaction of input triplets, we apply the same rationale as in §2.2 and consider a decomposition of the mutual information of an input triplet (*X*_1_, *X*_2_, *X*_3_) and the output (derivation provided in appendix A.3)

(2.8) $$I(X_{1},X_{2},X_{3};Y)=\underset{\text{first}-\text{order}}{\underset{︸}{I(X_{1};Y)+I(X_{2};Y)+I(X_{3};Y)}}+\underset{\text{second}-\text{order}}{\underset{︸}{I(X_{1};X_{2}|Y)+I(X_{1};X_{3}|Y)+I(X_{2};X_{3}|Y)}}+\underset{\text{third}-\text{order}}{\underset{︸}{I(X_{2};X_{3}|X_{1},Y)-I(X_{2};X_{3}|Y)}}.$$

Having identified the first- and second-order terms on the right-hand side, the decomposition suggests interpreting the remainder as the genuine third-order sensitivity measure. Using the notation *I*_3_, we define

(2.9) $$I_{3}(X_{1};X_{2};X_{3}|Y)\overset{\text{def}}{=}I(X_{2};X_{3}|X_{1},Y)-I(X_{2};X_{3}|Y).$$

This quantity is the conditional form of what McGill (1954) called ‘interaction information’. McGill also showed that interaction information is symmetric with respect to permutations of its arguments, meaning for the conditional form that

(2.10) $$I_{3}(X_{1};X_{2};X_{3}|Y)=I(X_{1};X_{2}|X_{3},Y)-I(X_{1};X_{2}|Y)=I(X_{2};X_{3}|X_{1},Y)-I(X_{2};X_{3}|Y)=I(X_{1};X_{3};X_{2},Y)-I(X_{1};X_{3}|Y).$$

Note that interaction information can be negative. By virtue of equation (2.10), this can only happen when all three pairwise interactions have non-zero conditional mutual information. Negative interaction information then indicates an inner redundancy of the triplet (*X*_1_,*X*_2_,*X*_3_), in the sense that the pairs (*X*_1_,*X*_2_), (*X*_2_,*X*_3_) and (*X*_1_,*X*_3_) do not provide entirely independent pieces of information about *Y*. This situation rarely occurs in natural systems, although appendix A.1 presents a contrived and artificial example with negative interaction information.

Generalizing the definition of *I*_3_, one can analogously quantify the fourth-order sensitivity via the fourth-order conditional interaction information

(2.11) $$I_{4}(X_{1};X_{2};X_{3};X_{4}|Y)=I(X_{1};X_{2}|X_{3},X_{4},Y)-I(X_{1};X_{2}|X_{3},Y).$$

Higher order interactions can be defined accordingly.

### 2.4 The information balance: a summation theorem for sensitivity indices

Having identified measures for first-, second- and higher order sensitivities, we consider a decomposition of the total mutual information for arbitrary number of inputs. Generalizing equation (2.3), one obtains the general form of the information balance for a system with *n* inputs

(2.12) $$I(X_{1},...,X_{n};Y)=H(Y)-H(Y|X_{1},...,X_{n}).$$

In addition to *H*(*Y*), which is straightforward to compute, the ‘noise entropy’ *H*(*Y*|*X*_1_, …, *X*_*n*_) has to be evaluated. In a deterministic system, this quantity vanishes for continuous random variables. However, computing information-theoretic quantities in a continuous fashion would require parametric models of all random variables. Since the input–output mapping is often not given in closed form, it will generally be impossible to derive such models analytically. Moreover, there is no general parametrization scheme that can be fitted to the multitude of possible empirical distributions arising in various systems. Rather, the parametric distributions must be selected on a case-by-case basis, with no guarantee of obtaining a close fit. In our experience, it proved very difficult to match the heavy-tailed output histograms arising in our particular application (cf. §3) with any standard distribution function.

Hence, to make the information-theoretic quantities measurable, we choose to discretize all variables. The noise entropy then takes the role of the *residual uncertainty* in *Y* that persists, given all inputs with a finite precision determined by the imposed discretization. We shall refer to this residual uncertainty as the *discretization entropy*, denoted by *H*_Δ._ In §3.4, we show how *H*_Δ_ can be estimated via Monte Carlo simulation.

Normalizing by *H*(*Y*)−*H*_Δ_—the maximum amount by which the output uncertainty can be reduced by the parameters—one can rewrite the information balance for a discretized deterministic system as

(2.13) $$\frac{I(X_{1},...,X_{n};Y)}{H(Y)-H_{\Delta }}=1.$$

Equation (2.13) provides the basis for a summation theorem, since it is possible to express the left-hand side in terms of the previously defined sensitivity indices, as shown in appendix A.3. Decomposition up to third order yields

(2.14) $$\frac{1}{H(Y)-H_{\Delta }}\left\{\sum_{i}I(X_{i};Y)+\sum_{i<j}I(X_{i};X_{j}|Y)+\sum_{i<j<k}I_{3}(X_{i};X_{j};X_{k}|Y)+\Delta I\right\}=1.$$

Here, summations extend over all index combinations excluding permutations. While related decompositions of the information of an ensemble of variables have been considered previously (Watanabe 1960; Fano 1961; Panzeri *et al*. 1999; Amari 2001; Schneidman *et al*. 2006), they have never been applied in the context of sensitivity analysis (see appendix A.1 for a discussion of alternative decompositions).

Calculation of the conditional mutual information requires knowledge of the underlying marginal and joint probability density functions. In practice, these densities must be estimated empirically by means of marginal and joint histograms. Particularly, the empirical estimate of a joint density can be problematic when the amount of available data is insufficient to populate all the bins in its joint histogram. This leads to a systematic error (‘bias’) in the limited-sampling estimation of information; the higher the dimensionality of the histograms to be sampled, the larger the bias. Thus, the estimation of higher order interactions is particularly difficult. However, reliable correction of the sampling bias is possible using advanced statistical techniques (Panzeri & Treves 1996; Nemenman *et al*. 2004; Montemurro *et al*. in press). Given the amount of simulations we could produce in the particular application presented below, these techniques allowed an accurate elimination of the bias for up to third-order interactions. Only the first-order quantification would have been possible without using such bias reduction techniques.

Even though, for the practical reasons described above, sensitivity indices can only be evaluated up to a certain order, the remainder Δ*I*—the combined effect of all higher order interactions—can be assessed since all other terms in the equation are known. If the lower order sensitivity indices capture the essence of the dependence structure, the remainder will be a small fraction of *H*(*Y*)−*H*_Δ_. A significant value of Δ*I* would indicate that important higher order interactions exist, which is generally not expected in most simple systems (Rabitz & Aliş 1999). In large networks, higher order interactions require an extreme number of connections, unless the degree of connectivity varies strongly across the network. Hence, one would expect to find a small number of local ‘hubs’ forming highly connected subnetworks. While this is still the subject of debate, we note that the complex networks arising in biological systems do indeed tend to have sparse intrinsic connectivity patterns (Wagner & Fell 2001; Barabási & Oltvai 2004; Csete & Doyle 2004).

### 2.5 Total sensitivity indices

A very useful concept in variance-based sensitivity analysis is the so-called *total sensitivity index* (Saltelli *et al*. 2005), which measures the overall influence that a particular input exerts on the output, comprising main effects and *all* interactions. In the ANOVA framework, the total sensitivity expresses the remaining output variance when all other inputs are kept fixed. The idea is to calculate this quantity without relying on the other sensitivity indices (first, second, third-order and so forth). If a total sensitivity index is zero, the corresponding input is irrelevant; if not, it is interesting to relate it to the other indices. For instance, comparing the total sensitivity index of an input with its first-order index reveals the degree to which the input is interacting with others.

This concept can be readily applied to information-based sensitivity analysis. The information-theoretic total sensitivity index for variable *X*_*i*_ is given by

(2.15) $$s_{\text{total},i}=\frac{H(Y|\{X_{1},\ldots ,X_{n}\}\backslash X_{i})}{H(Y)-H_{\Delta }}.$$

The total sensitivity index can also be expressed as the sum of all sensitivity indices involving *X*_*i*_:

(2.16) $$s_{\text{total},i}=\frac{1}{H(Y)-H_{\Delta }}\left\{I(X_{i};Y)+\sum_{\begin{array}{l} j\\ j\neq i \end{array}}I(X_{i};X_{j}|Y)+\sum_{\begin{array}{l} j,k\neq i\\ j<k \end{array}}I_{3}(X_{i};X_{j};X_{k}|Y)+\ldots \right\}.$$

Note, the sum of all total sensitivity indices is generally greater than 1 since expansions for different input variables will share certain sensitivity indices if the variables interact.

## 3. Information-theoretic sensitivity analysis of a model of the NFκB signalling pathway

As an example, we apply our methodology to parameter sensitivity analysis in systems biology. We consider a model of the (IκB)/NFκB signalling pathway (Hoffmann *et al*. 2002) and investigate the interdependencies among intrinsic parameters (in this case 64 reaction rate constants) with respect to their influence on the time course of the concentration of a particular metabolite, the nuclear transcription factor NFκB, which is a key component in early immune response.

In a nutshell, the pathway model works as follows. There are three main components: NFκB, IκB inhibitory proteins (IκBα and its isoforms IκBβ and IκBε) and the IκB kinase (IKK). The model describes the kinetics of interaction between these components, their transport between nucleus and cytoplasm, the inhibitor IκB degradation, as well as the NFκB-regulated gene expression and subsequent resynthesis of the inhibitors (figure 2). NFκB is normally bound in an IκB–NFκB complex. Following a step increase in the concentration of IKK, which models the effect of an extracellular stimulus (e.g. tumour necrosis factor (TNFα)), NFκB is released from the IκB–NFκB complex and enters the nucleus. The IκBs are rapidly degraded. In the nucleus, NFκB regulates the expression of genes leading to a resynthesis of the IκB inhibitor proteins. The newly synthesized IκB binds to the nuclear NFκB forming an IκB–NFκB complex and subsequently shuttles NFκB back to the cytoplasm, thus initiating a negative feedback loop. The cycle is repeated until all IKK has decayed. As a result of the delayed negative feedback, the concentration of nuclear NFκB exhibits an oscillatory behaviour (figure 3) that can be characterized in terms of features such as peak amplitude, frequency or phase (Nelson *et al*. 2004; Kell 2006*a*).

To exemplify the sensitivity analysis, we select one feature as output *Y*, namely the time difference between the first two peaks of the nuclear NFκB oscillation, *P*_1_=*T*_2_−*T*_1_. Evaluating the input–output function thus involves numerically solving a system of 24 ordinary nonlinear differential equations (ODEs) corresponding to the reaction equations and subsequently determining the first two maxima in one component of the solution. The entire analysis is performed with respect to the selected feature, based on 680 000 simulations, which yield sufficiently accurate information measures up to third order. All parameters were varied simultaneously, each drawn independently from a uniform distribution over the interval from 0.9 to 2.0 times the nominal value, and discretized into 15 bins. The lower bound of the sampling interval is dictated by the empirical observation that oscillations are only guaranteed to occur for parameter values above about 0.9 of the nominal values given in Hoffmann *et al*. (2002).

### 3.1 First-order sensitivity indices

The first-order analysis reveals that only a small subset of about eight parameters out of 64 (or at most 11, if one includes parameters 1, 19 and 37) significantly influence the output feature *P*_1_ (figure 4). The parameters thus identified either directly affect the amount of available NFκB (9, cytoplasmic release; 19, nuclear import; 1, IκBα–NFκB association) or control the strength of the negative feedback, which depends on the production of the inhibitor protein IκBα (28, transcription; 36, constitutive translation), its transport into nucleus (38, nuclear import of IκBα), its destruction (37, degradation; 29, IκBα mRNA degradation; 62, IKK–IκBα catalysis). In addition, the feedback is *indirectly* affected by the IκB kinase (IKK). Therefore, its decay rate (61) and the rate of binding IKK to the IκBα–NFκB complex (52), a step prior to the release of NFκB, are also important. Parameters specifically relating to the inhibitor isoforms IκBβ or IκBε are insignificant, which is in accordance with experiments indicating that only the knockout of IκBα is lethal (Gerondakis *et al*. 1999).

The result is consistent with previous local sensitivity analyses of a different output feature (Ihekwaba *et al*. 2004, 2005) and in accordance with global sensitivity analysis of the overall time course of the nuclear NFκB concentration (Yue *et al*. 2006).

### 3.2 Second-order sensitivity indices

At the level of pairwise interactions, a rather small number of relevant pairs emerge (figure 5). No significant synergies are observed, meaning that only those pairs wherein at least one partner is individually relevant have significant interactions. The predominant contributions are by pairs where both partners have significant individual impact. The degree of interactivity, that is the number of relevant pairs in which a parameter appears, varies strongly. For instance, parameter 29 (the IκBα mRNA degradation rate) seems to play the role of a ‘super parameter’, in the sense that it is involved in most, and the strongest, interactions. Note that pairs with very small yet statistically significant sensitivities are not visualized in the interaction matrix (figure 5), due to limited diagram resolution.

Statistical significance can be assessed via a bootstrap test, which involves repeated random shuffling of the parameter sampling sequences and recalculation of the conditional mutual information from these shuffled sequences. Several hundred repetitions produce a bell-shaped distribution of random sensitivity values, the mean and standard deviation of which characterize the range of ‘chance values’ of the particular sensitivity index under consideration. If the index calculated from the original non-shuffled data is two or three standard deviations above its bootstrap mean, it can be considered statistically significant.

While the particular parameter set identified as most relevant is very biologically plausible, the strongly varying degree of parameter interactivity is surprising. For instance, it is not obvious why the degradation of the IκBα mRNA transcript (parameter 29) should play such a predominant role when compared with transcription (parameter 28) and translation (parameter 36). In fact, it has been suggested (P. Paszek & M. R. H. White 2007, personal communication) that this result might be an artefact of the model. The arising debate illustrates the usefulness of a detailed sensitivity analysis as a means of highlighting potential design flaws in complex models.

### 3.3 Third-order interactions

The sparseness of the interaction structure continues at the third order (figure 6). It is again the combinations of individually relevant parameters that exhibit the strongest tripletwise interactions. The assessment of statistical significance is analogous to the procedure described in §3.2. All third-order indices are positive.

### 3.4 Monte Carlo estimation of discretization entropy

Let *n* be the number of system inputs. Assume a discretization scheme where each input range is partitioned into the same number of bins, denoted by *n*_bins._ Let *j*_1_, …, *j*_*n*_ be the bin indices of the inputs *X*_1_, …, *X*_*n*_, with *j*_1_=1, …, *n*_bins_; *j*_2_=1, …, *n*_bins_ and *j*_*n*_=1, …, *n*_bins_; and let Δ*x*_1_, …, Δ*x*_n_ be the corresponding bin widths. Then the bins are defined as

(3.1) $$\begin{array}{l} B_{j_{1}}=x_{1,\text{min}}+[(j_{1}-1)\Delta x_{1},j_{1}\Delta x_{1}]\\ \vdots \\ B_{j_{n}}=x_{n,\text{min}}+[(j_{n}-1)\Delta x_{n},j_{n}\Delta x_{n}]. \end{array}$$

The discretization entropy is the averaged conditional entropy of *Y*, for which uniformly distributed input values is simply

(3.2) $$H_{\Delta }={\left(\frac{1}{n_{\text{bins}}}\right)}^{n}\sum_{j_{1},\ldots ,j_{n}=1}^{n_{\text{bins}}}H(Y|X_{1}=x_{1}\in B_{j_{1}},\ldots ,X_{n}=x_{n}\in B_{j_{n1}}).$$

The summation extends over the total number of input bin combinations, which is (*n*_bins_)^*n*^. Since this number can be very large, equation (3.2) cannot be generally evaluated. However, Monte Carlo estimates can provide excellent approximations. Figure 7 shows the estimated discretization entropy as a function of the number of bin combinations used to compute the average. Only several hundred bin combinations are required to obtain a reliable estimate. For each bin combination, about 100 evaluations of the feature *Y* were performed to estimate the local conditional histogram from which the conditional entropy $H\left(Y|X_{1}=x_{1}\in B_{j_{1}},\ldots ,X_{n}=x_{n}\in B_{j_{n1}}\right)$ in equation (3.2) is computed. The figure can be understood as a consequence of the output/feature uncertainty being small, for reasonably fine input discretization. Hence, the conditional histograms approximating $p\left(Y|X_{1}=x_{1}\in B_{j_{1}},\ldots ,X_{n}=x_{n}\in B_{j_{n1}}\right)$ tend to have only very few bins with non-vanishing probability. In our case, the output is discretized into 15 bins out of which usually only one or two have non-zero counts, which can therefore be estimated properly with about 100 data. Thus, 600×100=60 000 evaluations provide a reasonably accurate estimate of the discretization entropy.

### 3.5 Information balance

The block diagram of the information balance (figure 8) shows that higher order interactions do contribute significantly to the total sensitivity. Moreover, only a small subset of parameter pairs and triplets interact significantly (figures 5 and 6), and we expect such sparse connectivity to continue at higher orders.

### 3.6 Total sensitivity indices

Total sensitivity indices consist of conditional entropies of the type *H*(*Y*|{*X*_1_, …, *X*_*n*_}\*X*_*i*_), which therefore can be estimated in a similar fashion to the discretization entropy, except that the value of the input *X*_*i*_ under consideration is allowed to vary over its entire range. All other inputs are evaluated within their bins, and the conditional entropy is again averaged over (in theory) all bin combinations. For the system under investigation, the Monte Carlo estimates exhibit a convergence comparable to that in figure 7.

Figure 9 shows the estimated total sensitivity indices of the eight most relevant parameters of the NFκB pathway model next to their first-order indices, revealing the different degrees of interaction. The diagram leads to two main conclusions. First, parameter 29 stands out in terms of its overall significance, since it has the strongest individual impact and also the highest degree of total impact, in the sense that almost 80% of the output uncertainty is removed by the information contributions of 29 and its interactions. Second, the fractional contribution of the interactions to the total sensitivity is higher in the other parameters, but, with the exception of parameter 36, their interaction impact (the difference of total and first-order sensitivity) is lower than that of 29.

A total sensitivity index equalling unity would indicate that the corresponding input quantity is ‘fully connected’, in the sense that it participates in *all* relevant interactions; sensitivity indices not involving this parameter would be irrelevant. For parameter 29, this is almost the case.

## 4. Conclusions

With the advent of advanced estimation techniques, mutual information has become a viable means of characterizing input–output interactions in complex networks. The framework developed in this paper lays the theoretical foundations for an information-theoretic sensitivity analysis that assigns credit or influence to input variables in terms of their overall contribution to a system's output entropy. However, our method is far more than a replacement of *analysis of variance* by *analysis of entropy*. The information-theoretic approach does not rely on implicit assumptions of normally distributed outputs and is easily generalized to include non-orthogonal input sequences. Moreover, the information-theoretic approach lends itself well to the analysis of systems with an intrinsically stochastic structure, such as biochemical reactions with small numbers of molecules (Wilkinson 2006). In this case, the noise entropy provides a combined representation of the output uncertainty due to discretization and all sources of intrinsic stochasticity.

## Figures and Tables

**Figure 1:**
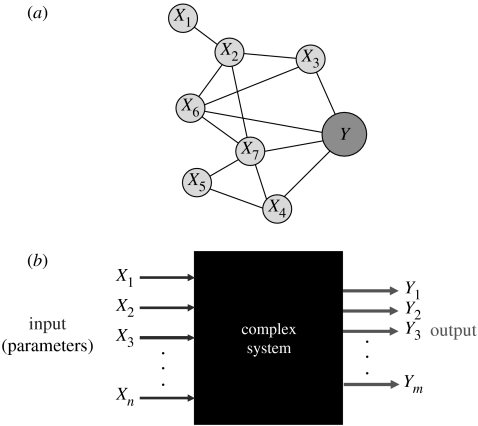
Complex systems with multiple inputs and outputs. This is a typical situation in systems biology. For instance, pathway models (*a*) are described by sets of coupled nonlinear ODEs (deterministic or stochastic). Input–output relations can only be elucidated by numerical evaluation of the system output, e.g. a flux, for various configurations of the input parameters. Global sensitivity analysis aims to determine the degree to which these inputs control the output, and how they interact. In most applications, the input–output mapping is nonlinear and not given in closed form; hence, the system is a ‘black box’ (*b*).

**Figure 2:**
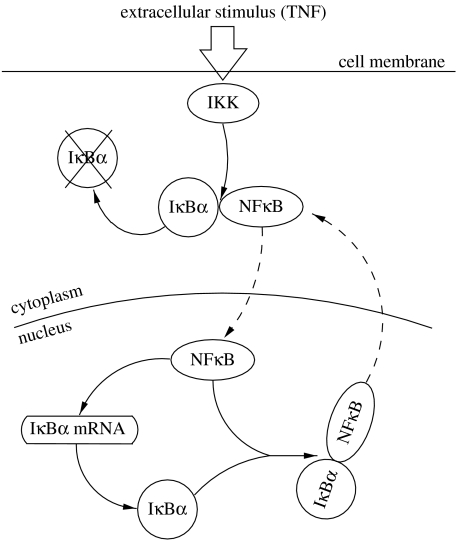
Schematic of the NFκB signalling pathway (isoforms of IκBα not shown). Solid arrows denote reactions and dashed arrows indicate translocation. In the model, an external stimulation suddenly raises the concentration of IKK, which catalyses the degradation of IκBα, leading to the release of NFκB that can then translocate to the nucleus. In the nucleus, NFκB regulates the expression of genes leading to a resynthesis of the IκBα inhibitor proteins. The newly synthesized IκBα binds to the nuclear NFκB forming an IκBα–NFκB complex, whereby NFκB is shuttled back to the cytoplasm thus initiating a negative feedback loop.

**Figure 3:**
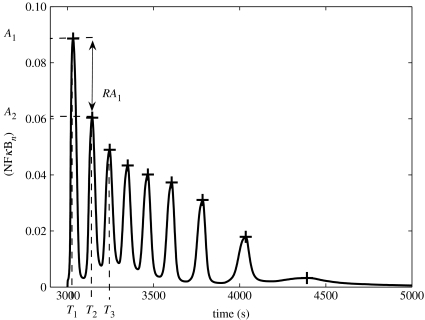
Example time course of the concentration of nuclear NFκB as determined by solving an ODE model. Characteristic output features include, among others, the timing *T* and amplitudes *A* of the peaks or functions thereof, such as *P*_1_=*T*_2_−*T*_1_. These features are considered to encode a signal that triggers subsequent cell fate via apoptosis or proliferation, for instance.

**Figure 4:**
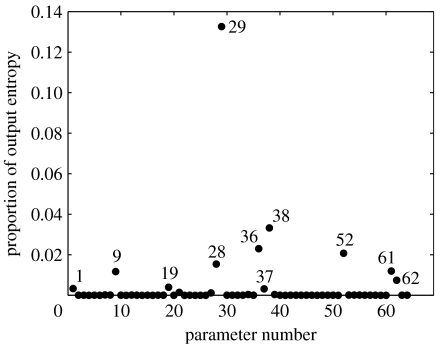
First-order sensitivity indices with respect to feature *P*1 (time difference between the first two maxima in the time course of nuclear NFκB concentration). Out of a total of *n*=64 parameters only a few exert significant influence on the feature.

**Figure 5:**
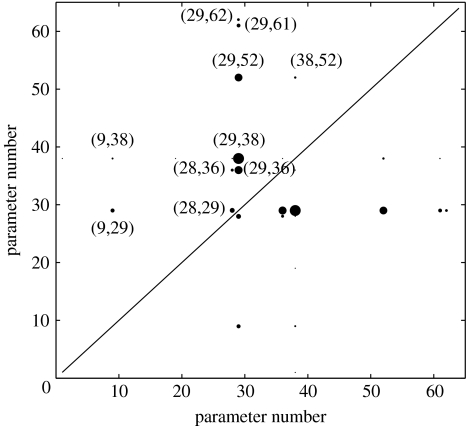
Second-order sensitivity indices in the form of an interaction matrix. The radius of the filled circles indicates the magnitude of sensitivity indices. The dependence structure is quite sparse. Only pairs with at least one individually relevant partner have significant interactions. The predominant contributions are by pairs where both partners have significant individual impact.

**Figure 6:**
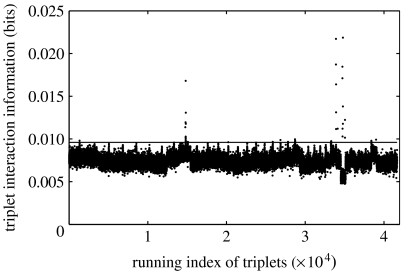
Third-order sensitivity indices. A sparse dependence structure is also found at the level of tripletwise interactions. The horizontal line marks the significance threshold (bootstrap mean plus 3 standard deviations, cf. §3.2).

**Figure 7:**
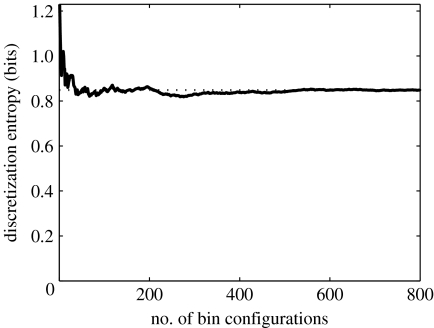
Example of a Monte Carlo estimate of the discretization entropy as a function of the number of bin configurations, computed for the feature *P*1 of the nuclear NFκB concentration. The graph shows a rapid convergence of the cumulative average over the regional output entropies computed from randomly selected input bin configurations, even though the total number of possible bin configurations (15^64^) vastly exceeds the number of actually sampled bins (up to 800). For each bin configuration, the input–output map was evaluated 100 times, which resulted in an accurate calculation of the corresponding conditional entropy.

**Figure 8:**
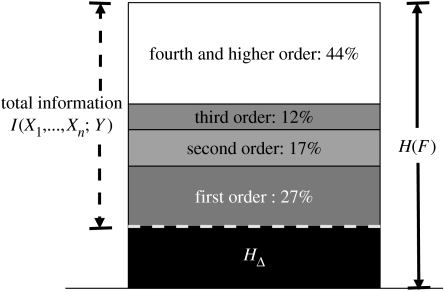
Block diagram of the information balance for a particular feature (*P*1) in the NFκB oscillation (cf. figure 3). The height of the entire block equals the output uncertainty (entropy). All contributions are normalized with respect to the total information, the amount of output uncertainty the inputs account for. The remainder *H*_Δ_ is the uncertainty due to the discretization of input values (c.f. §2.4). Obviously in this case, fourth- and higher order terms contribute a significant portion of the output entropy, indicating a high degree of parameter interaction. This result is supported by the high total sensitivity indices observed in the eight most significant parameters (cf. figure 9).

**Figure 9:**
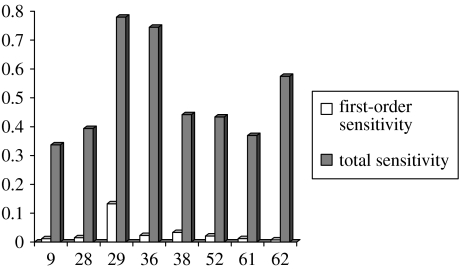
Comparison of first-order and total sensitivity indices for the most significant parameters with respect to feature *P*1 (cf. figure 3). The difference between the two measures indicates the parameter's degree of interaction. A total sensitivity value close to unity, as in parameter 29, indicates that the parameter and its interaction partners almost fully describe the system. Note, the total sensitivity indices do not sum to unity, unless there are no interactions, in which case first-order and total sensitivity indices are equal (additive system). In the example studied here, the interactions clearly dominate.

**Figure 10:**
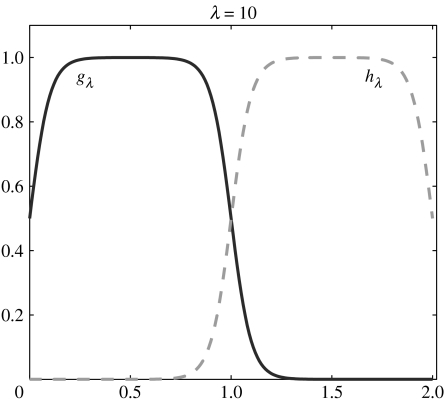
Graphs of the sigmoid pulse functions *g*_λ_ and *h*_λ_.

**Figure 11:**
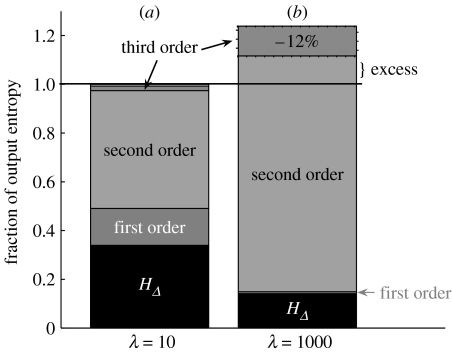
Schematic visualization of information-theoretic sensitivity indices by a block diagram. (*a*) Block diagram of an information balance with positive conditional interaction information (CII). Its components are the discretization entropy *H*_Δ_, the first-order sensitivity indices, the second-order indices and the third-order index given by the conditional interaction information. In this example, the CII is positive and hence an integral component of the output entropy, i.e. the sum of sensitivity indices is less or equal to the output entropy, with equality being reached once *all* relevant terms have been added. (*b*) Block diagram with negative CII. Here, the sum of discretization entropy plus first- and second-order terms exceeds the output entropy. The magnitude of the negative CII equals the information excess. The sum of all indices *does* equal the output entropy, but the sensitivity indices have different signs. In this case, an interpretation of the CII as a third-order sensitivity index is not meaningful, since the input pairs do not contribute independent pieces of information, yielding non-orthogonal information decomposition.

**Table 1:** Information balances of the example system defined in (A 1) for two different values of the control parameter *λ*. (*a*) Apart from a small numerical error, the sum of sensitivity measures equals the output entropy. (*b*) For large *λ*, the first-order terms are virtually irrelevant, whereas the second-order exceeds the output entropy corrected for discretization effects (*H*(*F*)−*H*_Δ_). This information excess is compensated by a negative third-order term.

	absolute information	fraction of *H*(*F*)−*H*_Δ_
(*a*) *λ*=10 (15 bins)		
*H*_Δ_	0.7257	
first order	0.3238	22.92%
second order	1.0338	73.17%
third order	0.0402	2.85%
sum	2.1235 bits	98.94%
*H*(*F*)	2.1385 bits	
(*b*) *λ*=1000 (15 bins)		
*H*_Δ_	0.1191	
first order	0.0067	0.92%
second order	0.8178	112.66%
third order	−0.1031	−14.20%
sum	0.8405 bits	99.38%
*H*(*F*)	0.8450 bits	

## Appendix A

### A.1 The sign of interaction information

In this section, we discuss the possibility of negative conditional interaction information (CII) and examine the conditions under which this can occur. Most maps do not seem to have this property, but we provide a carefully constructed example of a simple system with three parameters that can exhibit negative conditional interaction information. Consider the function

(A1) $$Y=f(X_{1},X_{2},X_{3})=g_{\lambda }(X_{1})g_{\lambda }(X_{2})g_{\lambda }(X_{3})+h_{\lambda }(X_{1})h_{\lambda }(X_{2})h_{\lambda }(X_{3}),$$

where $X_{1},X_{2},X_{3}\in [0,2]$, the components *g*_λ_ and *h*_λ_ are the functions

$$g_{\lambda }(x)=\frac{1}{2}\{\text{tanh}[\lambda x]-\text{tanh}[\lambda (x-1)]\},$$

and

$$h_{\lambda }(x)=\frac{1}{2}\{\text{tanh}[\lambda (x-1)]-\text{tanh}[\lambda (x-2)]\}.$$

Figure 10 shows a plot of the auxiliary functions. The control parameter *λ* determines the ‘steepness’ of the sigmoid components. For λ→∞, *g*_λ_ and *h*_λ_ become ‘square-wave pulses’. Symbolically, one can write

Hence, the random variables are weighted by indicator functions that respond to their values being either in the lower or upper half of their interval.

Figure 11 shows a schematic visualization of the two possible scenarios (different *λ*). If all the information-theoretic sensitivity measures capturing first-, second- and third-order effects have positive sign, these contributions—together with the discretization entropy—sum up to the output entropy (figure 11*a*). Under certain circumstances, the sum of first- and second-order indices exceeds the output entropy (figure 11*b*), where the pairwise interactions are not providing independent pieces of information. In this case, the excess information is compensated by a negative third-order contribution. Although the sum of all sensitivity indices does equal the output entropy, a meaningful interpretation of the components as second- or third-order sensitivity measures is no longer justified.

Table 1 shows the information balance of the system for two choices of *λ*. Owing to symmetry in the inputs, the information measures within each order of interaction are the same. Therefore, only the sums of first- and second-order terms are provided.

Apparently, the combination of non-monotonicity and point symmetry of the system (A 1) leads to the negative information measure. Choosing a smaller value of the control parameter *λ* destroys the symmetry, and consequently the CII becomes positive. Exact point symmetry is not likely to be a feature of natural systems such as our example from systems biology, since it would require an extreme degree of regularity. Therefore, we conclude that the example presented is a rare exception.

One potential alternative scheme that could provide an information decomposition based solely on non-negative quantities is the maximum entropy approach (Jaynes 1957; Amari 2001; Schneidman *et al*. 2006). For instance, Amari's elegant method of information geometry (Amari 2001) requires the calculation of surrogate maximum entropy distributions, referred to as *p*^(2)^_,_ *p*^(3)^, etc. Here, *p*^(2)^ has the same pairwise marginals as the joint density (*p*^(3)^, the same tripletwise marginals) but contains no higher order correlations. The Kullback–Leibler divergence of *p*^(2)^ and *p*^(1)^ can be taken to represent the total entropy attributable to second-order interaction and so on. However, at present, the maximum entropy approach is only fully developed with respect to a decomposition of the joint entropy of a set of variables (Amari 2001; Schneidman *et al*. 2006). We are currently investigating how to extend this approach to obtain a decomposition of the total information for an input–output relation. One practical concern in using a maximum entropy formalism for sensitivity analysis of complex systems with tens of parameters is that the maximum entropy method is (at present) computationally prohibitive and data-intensive. The maximum entropy densities are high-dimensional and suffer from a larger sampling bias than our approach, which is based on pair- and tripletwise contributions that can easily be corrected for the bias. In addition, in order to become useful for sensitivity analysis, the maximum entropy methodology needs to be extended to provide an information decomposition that *explicitly* identifies the particular most relevant parameter interactions.

Future research will also be directed at further illuminating the relation between properties of the input–output map (e.g. monotonicity) and the sign of higher order sensitivity indices.

### A.2 Bias corrected estimates of mutual information

Mutual information *I*(*X*;*Y*) between an input variable *X* and an output variable *Y* is a functional of the probability densities of input and output. In practice, these probabilities are usually not known *a priori* and can only be estimated empirically from a limited number *N* of independent joint observations (‘trials’) of *X* and *Y*. The statistical errors made in measuring the probabilities owing to limited sampling leads to a severe systematic error (bias) in the information measures. This section is devoted to explain how we corrected the bias problem. For brevity, we focus on *I*(*X*;*Y*), which we shall simply refer to as *I*, but these considerations would straightforwardly apply to other information quantities used in this paper, such as the conditional mutual information. For the sake of explanation, we suppose that *X* is an *n*-dimensional variable *X*={*X*_1_, …, *X*_*n*_}.

The bias due to sampling with *N* trials is defined as the difference between the limited sampling average value of information 〈*I*〉_*N*_ (〈…〉_*N*_ being a probability-weighted average over all possible (*X*, *Y*) outcomes with *N* trials) and the true value of information *I*. Subtracting the bias from the limited sampling estimate allows for a much more accurate estimation of the true information *I*. We observe that *I*(*X*;*Y*) can be written as the difference between two entropies

I(X;Y)=H(X)−H(X|Y).

The bias of *I* is the difference between the biases of the two entropies. It is well known (Miller 1955) that entropies are biased downward (i.e. their bias is negative). *H*(*X*) depends only on the marginal distribution *p*(*X*). Its bias is much lower than that of *H*(*X*|*Y*), which depends on *p*(*x,* *y*), which is obviously much harder to sample than *p*(*x*). As a result, *I* is biased upward. Analytical considerations (Panzeri & Treves 1996) show that the bias decreases approximately linearly when increasing the number of trials, and increases approximately exponentially when increasing the number of dimensions *n* of the *X*-space. This makes it difficult to estimate *I* for large *n*.

Fortunately, the bias of the entropy can be computed approximately and eliminated by means of a number of techniques. In our experience, one of the most effective is the Bayesian technique of Nemenman *et al*. (2004), which uses a family of prior distributions that are weighted to produce a uniform expectation of entropy before any data are sampled. As (*X*, *Y*) data become available, the entropy estimation is updated as an average over all the possible hypothetical probability distributions weighted by their conditional probability given the data. The algorithm converges to the true value of entropy quite rapidly with the number of trials *N*. Unless the dimensionality of the space *n* is too large, residual errors left after this bias reduction are small. Since the uncorrected estimate of *I* is biased upward, residual errors in the estimation of *I* tend to be upward as well (Montemurro *et al*. in press).

To check whether any residual error is small, we have developed a different way to estimate information that is biased downward rather than upward (Montemurro *et al*. in press). This allows one to check the reliability of information-based sensitivity measures by assessing the proximity of the upper and lower bounds. To produce a downward-biased estimate of *I*, we used the following procedure (Montemurro *et al*. in press). We considered the entropy that would be obtained if the input was independent at fixed output, that is *p*(*x*|*y*)=*Π*_*i*_*p*(*x*_*i*_|*y*). The corresponding entropy of this ‘independent’ distribution is called *H*_ind_(*X*|*Y*) and typically has a very small bias because only marginal probabilities have to be sampled.

Alternatively, correlations between input variables can be removed by ‘shuffling’ the data at fixed *Y*, thus creating pseudo *X*-vectors obtained by randomly combining *x*_*i*_ values from different trials in which the value *Y* was observed. The resulting entropy, called *H*_sh_(*X*|*Y*), has the same asymptotic value of *H*_ind_(*X*|*Y*) for an infinite number of trials, but has a much higher bias than *H*_ind_(*X*|*Y*) for finite *N*. Following the mathematical analysis of Panzeri & Treves (1996), Montemurro *et al*. showed that the bias of *H*_sh_(*X*|*Y*) is of the same order of magnitude as the bias of *H*(*X*|*Y*) but typically slightly larger. This observation suggests computing *I* in the following way:

$$I_{\text{sh}}=H(X)-H_{\text{ind}}(X|Y)+H_{\text{sh}}(X|Y)-H(X|Y).$$

Owing to the bias cancellation created by the entropy terms added to the r.h.s., *I*_sh_ has the same value of *I* for an infinite number of trials, but a much smaller bias for finite *N*. Moreover, since *H*_sh_(*X*|*Y*) is more downward biased than *H*(*X*|*Y*), the resulting bias of *I*_sh_ is negative (Montemurro *et al*. in press). Thus, in cases when the upward-biased estimator *I* and the downward-biased estimator *I*_sh_ coincide, we can be confident that our information estimate is unbiased. If they do not coincide, their difference provides an idea of the order of magnitude of our uncertainty in the information estimation.

### A.3 Decomposition of the total mutual information

The following three equations are elementary formulae, proofs of which can be found in standard textbooks (e.g. Cover & Thomas 2006):

(A2) H(X,Y)=H(X)+H(Y|X),

(A3) I(X;Y)=H(X)−H(X|Y)=H(Y)−H(Y|X),

(A4) I(X;Y)=H(X)+H(Y)−H(X,Y).

We next derive a decomposition of the total mutual information of three variables, which will serve as an auxiliary formula in the general decomposition with an arbitrary number of variables.

#### A.3.1 Decomposition with three variables

By means of theorem (A 4), the total mutual information of a pair of random variables (*X*, *Y*) and a third variable *Z* can also be expressed in terms of entropies:

I(X,Y;Z)=H(X,Y)+H(Z)−H(X,Y,Z)=H(X)+H(Y|X)+H(Z)−H(X,Y,Z)=H(X)+H(Y)−I(X;Y)+H(Z)−H(X,Y,Z),

where we have applied (A 2) in the second step and (A 3) in the third. Expressing *H*(*X*) and *H*(*Y*) in terms of mutual information with respect to *Z* by means of (A 3) we obtain

$$I(X,Y;Z)=I(X;Z)+H(X|Z)+I(Y;Z)+H(Y|Z)+\underset{︸}{H(Z)-H(X,Y,Z)}-I(X;Y).$$

Rearranging terms and using the relation *H*(*Z*)−*H*(*X*, *Y*, *Z*)=−*H*(*X*,*Y*|*Z*) yields

$$I(X,Y;Z)=I(X;Z)+I(Y;Z)+\underset{I(X,Y|Z)}{\underset{︸}{H(X|Z)+H(Y|Z)-H(X,Y|Z)}}-I(X;Y),$$

where by virtue of (A 4) the remaining entropy terms amount to the conditional mutual information of *X* and *Y* given *Z*.

Hence,

(A5) I(X,Y;Z)=I(X;Z)+I(Y;Z)+I(X,Y|Z)−I(X;Y).

By conditioning on a fourth variable *W*, one also obtains

(A6) I(X,Y;Z|W)=I(X;Z|W)+I(Y;Z|W)+I(X,Y|Z,W)−I(X;Y|W).

The decomposition (A 5) has previously been applied in computational neuroscience (Adelman *et al*. 2003).

#### A.3.2 Decomposition with arbitrary number of variables

The general decomposition with an arbitrary number of input variables can be derived by repeated application of (A 5) and (A 6). Assuming independent inputs *X*_1_, …, *X*_*n*_, the total mutual information can be expanded using (A 5) by setting *X*=*X*_1_ and *Y*=*X*_2_, …, *X*_*n*_:

$$I(X_{1},\{X_{2}\ldots ,X_{n}\};F)=I(X_{1};F)+I(X_{2},\ldots ,X_{n};F)+I(X_{1};X_{2},\ldots ,X_{n}|F)-\underset{=0,\;\text{if}\;\text{independent}}{\underset{︸}{I(X_{1};X_{2},\ldots ,X_{n})}}=\underset{\text{first}-\text{order}\;\text{term}}{\underset{︸}{I(X_{1};F)}}+I(X_{2},\ldots ,X_{n};F)+I(X_{2},\{X_{3},\ldots ,X_{n}\};X_{1}|F).$$

Thus, a first-order term has been separated. Applying (A 6) to the last term separates a second-order term

$$I(X_{1},X_{2}\ldots ,X_{n};F)=I(X_{1};F)+\underset{\text{second}-\text{order}\;\text{term}}{\underset{︸}{I(X_{1};X_{2}|F)}}+I(X_{2},\ldots ,X_{n};F)+I(X_{3},\ldots ,X_{n};X_{1}|F)+I(X_{2};X_{3},\ldots ,X_{n}|X_{1},F)-I(X_{2};X_{3},\ldots ,X_{n}|F).$$

Expanding the last two terms, again using (A 6), produces a third-order term

$$I(X_{1},X_{2},\ldots ,X_{n};F)=I(X_{1};F)+I(X_{1};X_{2}|F)+I(X_{2},\ldots ,X_{n};F)+I(X_{3},\ldots ,X_{n};X_{1}|F)+\underset{=I_{3}(X_{1};X_{2};X_{3}|F),\;\text{third}-\text{order term}}{\underset{︸}{I(X_{3};X_{2}|X_{1},F)-I(X_{3};X_{2}|F)}}+I(X_{4},\ldots ,X_{n};X_{2}|X_{1},F)-I(X_{4},\ldots ,X_{n};X_{2}|F)+I(X_{3};X_{4},\ldots ,X_{n}|X_{2},X_{1},F)-I(X_{3};X_{4},\ldots ,X_{n}|X_{2},F)-I(X_{3};X_{4},\ldots ,X_{n}|X_{1},F)+I(X_{3};X_{4},\ldots ,X_{n}|F).$$

Similarly, the remaining terms can be expanded further using auxiliary formulae (A 5) and (A 6), thus splitting up more and more terms of ever higher order, and yielding all possible combinations of input variables for each order.
